# Development of a 3D printer using scanning projection stereolithography

**DOI:** 10.1038/srep09875

**Published:** 2015-04-23

**Authors:** Michael P. Lee, Geoffrey J. T. Cooper, Trevor Hinkley, Graham M. Gibson, Miles J. Padgett, Leroy Cronin

**Affiliations:** 1 School of Chemistry, University of Glasgow, G12 8QQ, UK; 2 School of Physics and Astronomy, SUPA, University of Glasgow, G12 8QQ, UK

## Abstract

We have developed a system for the rapid fabrication of low cost 3D devices and systems in the laboratory with micro-scale features yet cm-scale objects. Our system is inspired by maskless lithography, where a digital micromirror device (DMD) is used to project patterns with resolution up to 10 µm onto a layer of photoresist. Large area objects can be fabricated by stitching projected images over a 5cm^2^ area. The addition of a z-stage allows multiple layers to be stacked to create 3D objects, removing the need for any developing or etching steps but at the same time leading to true 3D devices which are robust, configurable and scalable. We demonstrate the applications of the system by printing a range of micro-scale objects as well as a fully functioning microfluidic droplet device and test its integrity by pumping dye through the channels.

The transformation of bespoke designs into real world objects is becoming commonplace in prototyping and development as reasonably priced commercially available 3D printers produce objects with high precision. However, much of the hoped for scientific advances require design features well below 1 mm. Such structures are envisioned for tissue engineering, whereby cells are be positioned within a scaffold^1^,^2^, as microfluidic devices^3^,^4^, or as customised precision labware, such as optics components. In general, various options exist for fabrication processes which avoid the need for expensive masks^5^,^6^,^7^,^8^,^9^. 3D printing is used widely in manufacturing industries, but has recently been taken up as a research tool. Commercially available 3D printers have already been used to cheaply fabricate devices with sub-millimetre features^10^,^11^,^12^. However, as a research tool they can lack the flexibility needed in research laboratories. There are two complementary development streams for 3D printing, namely fused deposition modelling (where parts are built by extruding heated plastic) and stereolithography (where a scanning laser polymerises a photoresist).

Within the stereolithography paradigm, Lu et al. incorporated a digital micromirror device (DMD) into the optical path of the laser, which replaced the need for scanning, and allowed a complete layer to be built with a single exposure^13^,^14^,^15^,^16^,^17^,^18^. DMDs, consisting of hundreds of thousands of individually addressable moving micromirrors, were originally developed for the display industry^19^, but have also found applications in other areas including wavelength multiplexing^20^, hyperspectral imaging^21^ and computational imaging^22^. They offer a method of spatially modulating light which is fast, highly efficient and works over a broad range of wavelengths. An array of micromirrors controls the reflection path of light in an optical system according to which ever image is displayed: each pixel of the image corresponds to an individual micromirror which switches its orientation between +/−12 degrees to the beam axis. For the purposes of stereolithography, this means that only the desired regions are exposed and cured, with the x−y resolutions ultimately being given by the size of the pixel of the DMD (and the z resolution given by the layer thickness of the photopolymer). In a further adaption of stereolithography, it was shown that stitching multiple patterns was possible by reducing the intensity of the perimeter of an pattern and exposing them twice (for the edges) or four times (for the corners). The photoresist sample was mounted on an automated stage which positioned the projection between exposures^7^.

Herein, we present a low cost system (built for under $3000) that utilises a stitching procedure but also incorporates an axial stage for controlling the height of the build platform in a tank of photopolymer^23^,^24^. This allows entire, large-scale 3D devices to be fabricated in the bulk fluid without any intermediate processing steps. The comparatively low cost is achieved through in house construction of most parts, including stages. We use 3D models of microfluidic devices (complete with tube connection ports) and compute a series of layers from which to build the device. If the layer is too large for a single projection, it is divided into smaller, overlapping projections which are displayed sequentially on the DMD. It is the combination of lateral stitching with the layer by layer building of components which gives a scalable, speedy technique which is capable of producing feature sizes that are comparable with microfluidic systems. To illustrate the potential of our system, we investigate the parameters affecting the resolution of the printed devices and demonstrate the system by printing a simple microfluidic structure. We show that these devices are viable microfluidic structures by connecting one to a syringe and pumping aqueous and oil-based dye through the channels.

## Results

### Characterisation of system

Calibration of the system is twofold. First, we need to map the size of the DMD pixels to the size of the projected image, and second, we need to calibrate the microsteps of the three axes stepper motors. The image size was calibrated by exposing a single image featuring a grid pattern and measuring the grid (in both x and y) through analysis of images acquired by a Keyence digital microscope. From this measurement, we can determine the distance (in mm) needed to move the projector for stitching in x and y. A calibration of the microsteps to millimetre was obtained by attempting a stitched structure with an estimate of this calibration, and measuring the error in the structure. This measurement was again performed by analysing images taken by the Keyence digital microscope. The z calibration (microsteps to millimetres) was obtained by printing a stack of images with an estimated calibration and measuring the error in that estimate with the Keyence digital microscope.

**Figure 1:**
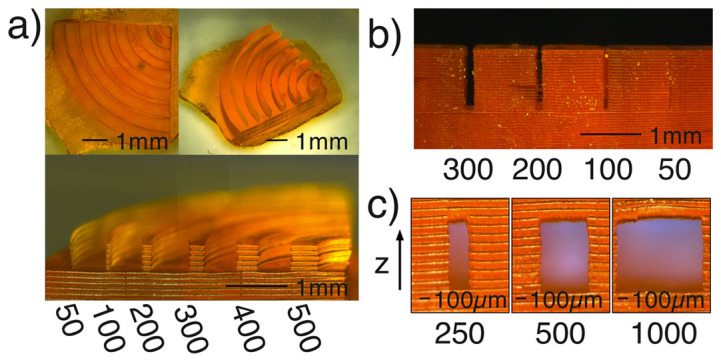
Images in (a) show a plan, isometric and end elevation view, of a characterisation structure. The structure consists of concentric curved wall so that we can observe the x and y lateral resolution. The walls have different thicknesses as indicated (all values in µm) and are separated by 500µm. The layer thickness is 100µm. In (b) we measure the narrowest channels we can print. The structure has a depth of 5mm, and layer thickness of 100µm. The 50µm channel is blocked, the 100µm channel is partially open, the 200 µm is mostly clear, while the 300µm channel is fully open. In (c) we examine the capability of the printer at printing horizontal overhanging structures, which is important when designing fluid channels.

To characterise the system we print test patterns featuring progressively smaller features. First we print vertical walls with varying thickness. The walls are curved in a quarter circle so we can observe both x and y characteristics. Results are shown in Fig. 1 a). We found that 50 µm walls were printable, and that there was no obvious discrepancy between x and y. Each layer is set at 100µm, which defines the limit of the axial resolution. The lateral resolution is ultimately limited by the pixel size of the DMD. If higher resolution is desired, it may be possible to change the imaging optics to demagnify the image of the DMD on the sample. Conversely, if large objects are required, we could adjust the magnification in order to reduce the number of exposures (and hence reduce build time). In this work, we have approximately a 1:1 imaging system meaning the minimum exposable area is around 10µm^2^. In practice we require an area of 50µm^2^ exposed to polymerise the monomer with the laser due to diffusion of the free radicals. In future work, we aim to optimise the chemistry such that diffusion of the free radicals is minimised and resolution improves^25^. Limiting diffusion will also enhance printing of channels, where we found the gap between walls to be limited to around 200µm.

### Example structures

**Figure 2:**
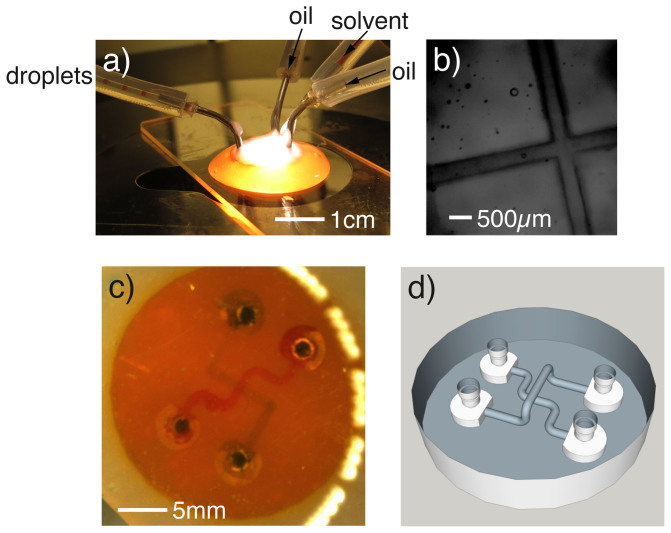
Fluidic devices. (a) and (b) show a droplet device. The channels are 400 µm in diameter and the device has around 1cm^2^ footprint and is 2.3 mm tall. In (a), there is a photograph of the device in operation, outputting droplets on the left. In (b) we show a microscope image of the channels within the device. (c) is a micrograph of a fly-over channel, where red and blue aqueous dyes cross over one another. (d) shows the 3D model of the fly-over device.

**Figure 3:**
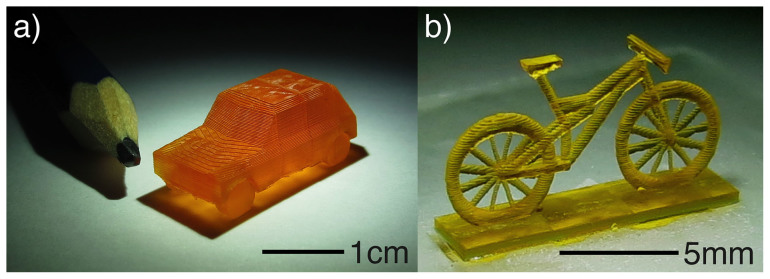
Images of printed miniaturised structures. (a) Shows a photograph of a automobile structure with the nib of a pencil shown alongside for scale (the length of the structure is 2 cm. The layers are 100µm and there are 3200 overlapping exposures in total. The print copes well with the severely overhanging chassis (no support material was used). (b) Shows a model of mountain bike printed with 100µm layers. The printer is capable of the fine detailed spokes on the wheels which are 200µm in diameter.

Figure 2 a) and b) shows an example millifluidic device fabricated using our system. As can be seen, the entire device is printed as a single unit. Connection tubing is push fitted to specially designed connection ports then sealed using epoxy glue. The device was therefore robust to leaks when a methanol or water solution of dye was used. The channels used here had a 400µm cross-section, and was capable of producing droplets consistently. Despite the Sudan I opaquing agent and the epoxy seal, we were able to obtain a reasonable observation of the channels with an Olympus IX81 microscope, Fig. 2 b). We also printed flow mixing devices and three dimensional networks of channels including channels passing over one another (see Supplementary Figs. S1–S5). The printer is not limited to fluidic devices, and in Fig. 3 a we show images of a miniaturised model measuring 2cm by 1cm by 1cm and consisting of 3200 stitched exposures, i.e., a Mini made by our “Minimaker” system.

**Figure 4:**
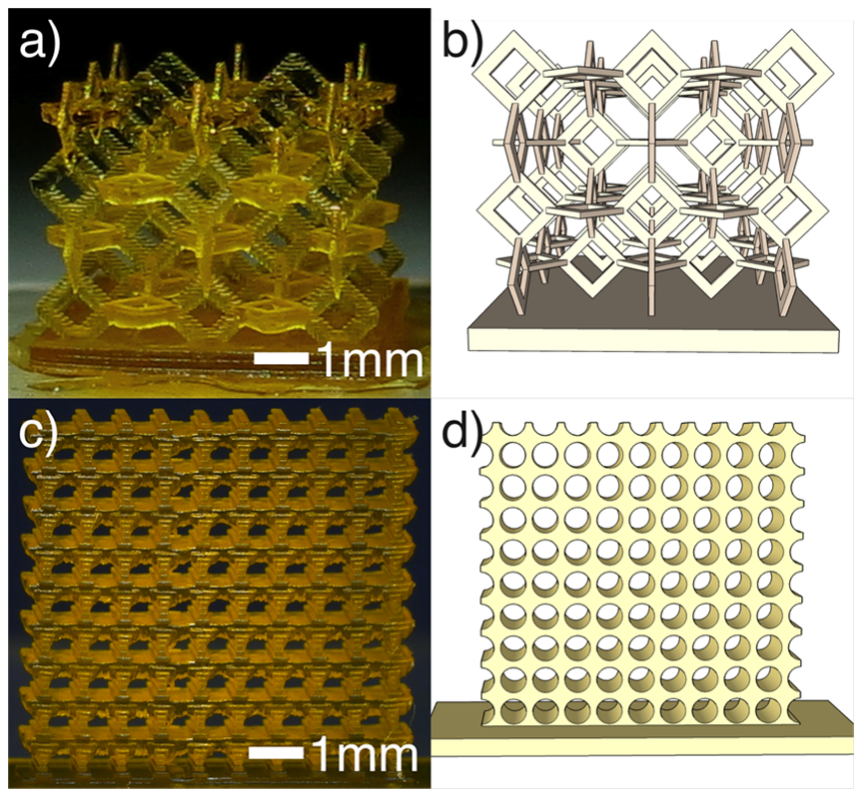
The printer was used to print intricate geometries. (a) and (b) show, respectively, a photograph and 3D model of a square lattice structure while (c) and (d) show a square array of circular holes (photograph and model, respectively).

3D printing has recently been used to create structures with interesting mechanical properties. A future application of our printer could be to explore such materials and in Fig. 4 we demonstrate the viability of the printer to create intricate structures. Figure 4 a) shows a structure created from a 3D lattice of squares at a 45° angle, accompanied by a 3D model, (Fig. 4 b). Figure 4 c) shows a square array of holes, a shape which has been shown to demonstrate buckling instability under compression^26^,^27^. While we do not perform any analysis of the mechanical properties of these objects, which requires further development of the photo polymer, we show that the printer is in principle capable of making these components.

## Discussion

The convergence of microfluidic and 3D printing technologies has the potential to provide rapid fabrication of custom devices with complex structures compared to 2D fluidic system. Currently, the resolution of low cost 3D printers is improving rapidly.

We have designed and built a UV stereolithographic 3D printer incorporating a digital light projector acting as a programmable mask and custom precision stages equipped with Hall effect sensors which are capable of positioning the stage to 1µm (Supplementary Fig. S6). A range of calibrations were performed, including imaging, stage movement, exposure time and layer height (Supplementary Figs. S7–S9). The smallest features we were able to print were 50µm walls, however, channels were limited to 200µm owing to diffusion of the polymer. These parameters are shown to be similar in the axial direction. The printing procedure involves stitching exposures together which enables large structures to be fabricated with high resolution. To demonstrate this, we printed a centimetre-scale object requiring the accurate stitching of thousands of exposures. We also printed a microfluidic device which was used to create microliter droplets of aqueous dye in oil. The flexibility of having built most of the components from scratch affords new and interesting avenues of explorations in microfluidics and micro-structuring.

## Methods

**Figure 5:**
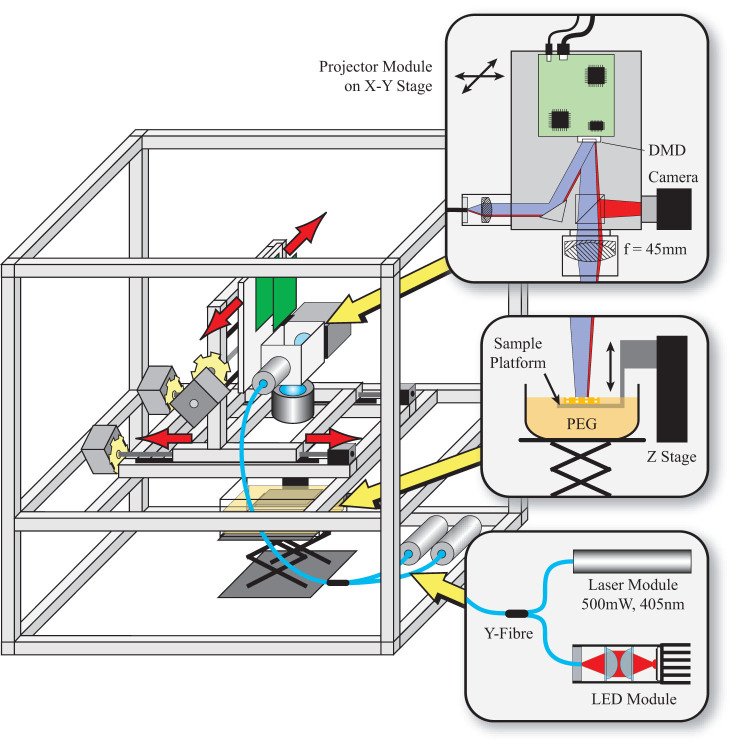
Schematic of the system. An aluminium frame is used to support the custom precision translation stages and projector system. Light from a UV laser and a red LED are combined using a Y-fibre, which is then used to illuminate a Digital Micromirror Device (DMD) projector. A lens is used to project the image of the DMD onto a sample slide which is which is immersed in the photopolymer.

A schematic of the optical system is shown in Fig. 5. A diode laser module (405nm, 500mW) (for writing) and a red LED (Luxeon Rebel) (for illumination) are coupled into separate channels of a Y-fibre, resulting in a combined output at the fibre end. The output is then collimated and used to illuminate a DMD (Texas Instruments, LightCrafter with 0.3WVGA chipset). The LED illumination and projection optics have been removed from the LightCrafter to allow direct access to the DMD chip, consisting of 608 × 684 pixels of pitch = 7.6µm, arranged in a diamond geometry. We image the DMD chip directly on to the printing stage with a lens, such that the total area for one exposure is 4 mm by 2 mm. Each exposure has a three second duration, which is sufficient to polymerise the photopolymer. The same lens that is used to focus the DMD pattern is also used to image the reflected light on to a CMOS camera chip (Prosilica, GC640M) positioned in an image plane of the DMD. The camera is used for vertical alignment, prior to each print. The UV laser is switched off and the z-stage is positioned such that the image formed by light from the red LED is in focus. The use of the red LED illumination avoids accidental curing of the photopolymer during set up.

The optical throughput of the system depends on various factors including the reflectivity of the individual micromirrors, the fill factor and transmission and reflective losses at the window of the DMD device. We measure the optical power reaching the DMD to be 60mW, giving an estimated power density of 20mW/cm^2^ at the sample. The optics module is mounted on a custom x−y stage constructed from aluminium profile.

Stepper motors control the x and y scan directions, scanning the optics module while the sample stage remains fixed. A separate stepper motor and linear stage controls the height of the sample in the photopolymer tank. After each layer is printed, the stage is lowered by 100 µm to allow a new layer of photopolymer to cover the surface of the object. In order to ensure the subsequent layer is exactly on top of the previous layer, there are Hall Effect sensors on the x and y axis of the stage and neodymium magnets fixed on the frame. When used in conjunction, this gives a reliable definition of the x−y origin, from which point the proceeding layer can be built. We always begin the next layer by approaching the origin from the far side in order to remove backlash from the stepper motors. The x, y and z axis stepper motors are controlled using a USB to serial communications link to the control electronics. A similar communications link controls the 405nm laser module, allowing the software to switch the laser on and control the exposure time.

The photoresist comprises a monomer: poly(ethylene glycol) diacrylate (Mn250) with three additives dissolved in it: a photo activator: phenylbis(2,4,6-trimethylbenzoyl)phosphine oxide (47.8 mmol), an opaquing agent: Sudan I (2.0 mmol) and a surfactant: Triton X-100 (0.5 mmol). The photo activator is sensitive to UV light at 405nm and when exposed, forms highly reactive radical species which initiate a chain-reaction cross-linked polymerisation of the monomer (see Supplementary Fig. S10 for a scheme of the reaction). Sudan I acts as an opaquing agent and absorbs UV light without reaction. This limits the depth to which the UV light can penetrate the photoresist solution and thereby ensuring that a good z-resolution is maintained even when printing overhanging structures. The purpose of Triton-X is to modify the surface tension of the solution and ensure that an even coverage of the next layer is achieved rapidly when the stage is lowered.

**Figure 6:**
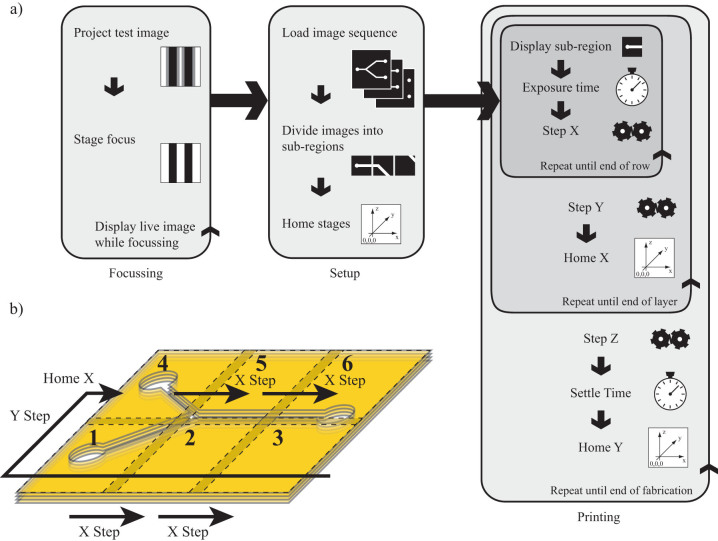
Fabrication procedure. Part (a) shows the flow diagram for initial set up and printing, while (b) shows an oblique view of a representation of how a print is built.

The control software, including the user interface, was developed using LabVIEW (National Instruments) and Python. The software has three functions. First, it creates the exposure sequence from a 3D model chosen by the user. Second, it operates the laser and the x, y and z stages. Finally, it reads and displays camera images of the print. For the exposure sequence, an STL file of a 3D structure is selected, from which a sequence of horizontal slices is calculated in Python. Each slice is converted into an 8-bit PNG image file whereby white (or 255) corresponds to areas of structure, and black (0) corresponds to void. The images are then divided (in LabVIEW) into a sequence of smaller images that will be stitched together during the print, Fig. 6b).

To avoid overexposure of the stitched regions, the grayscale values of overlap along the edges of adjacent images are multiplied by 0.7. When four images overlap at the corners the grayscale values are multiplied by 0.5, see Supplementary Figs. S11–S13. The stage and laser control software (Supplementary Figs. S14 and S15) is used during a print and in the initialisation of the printer. For the initialisation, a manual stage supporting the photopolymer tank is raised then lowered in order to dip the stage in the photopolymer. A frosted glass slide is placed on the stage and exposed to the curing laser for a few seconds which sets the photopolymer between the slide and stage. This is in order to fix the coverslip to the stage. We use frosted glass as we found prints adhere more readily to it (i.e., objects do not lift off during fabrication). Coupled with this, the initial layer of the print is exposed for 20 seconds which ensures the print sticks to the slide.

For the axial alignment of the print, photoresist is raised up beyond the stage and positioned such that the DMD is imaged exactly on the surface, as verified by the observation camera. The stage is then raised up until surface tension distorts the image acquired by the camera. This means that a thin layer of photoresist is on the print slide initially and that subsequent layers will be printed in focus.

The print begins by first “homing” the stages, then exposing the first pre-calculated image and moving the projector module one projection's width. At the end on the row, the stage is returned to home in x then moved in y. Our homing precision was measured to be 1µm This sequence continues until the final row where the z-stage is moved one layer down, into the photoresist, then the stages are homed, allowing time for the new layer of the photopolymer to flow over the print, Fig. 6.

## Author Contributions

L.C. and M.J.P. conceived the idea, planned experiments and wrote the paper with M.P.L., G.M.G. and G.J.T.C. T.H. developed the software, G.J.T.C. developed the chemistry, G.M.G. and M.P.L. developed the optics, and M.P.L. and G.J.T.C. designed and printed the structures. M.P.L., G.J.T.C., T.H. and G.M.G. jointly built and tested the system.

## Supplementary Material

Supplementary Information SI data
