# Supplementary material for: Development of a 3D printer using scanning projection stereolithography
Source: Sci Rep. 2015 Apr 23;5:9875. doi: 10.1038/srep09875 (PMC4407727; doi:10.1038/srep09875)
Supplement: Supplementary Information — SI data [file srep09875-s1.pdf]

Development of a 3D printer using scanning projection stereolithography – Supplementary information

Michael P. Lee[1], Geoffrey J. T. Cooper[1], Trevor Hinkley[1], Graham M. Gibson[2], Miles J. Padgett[2,*], and Leroy Cronin[1,]**

[1] School of Chemistry, University of Glasgow, G12 8QQ, UK
[2] School of Physics and Astronomy, SUPA, University of Glasgow, G12 8QQ, UK
* miles.padgett@glasgow.ac.uk;
**leroy.cronin@glasgow.ac.uk

Additional Prints

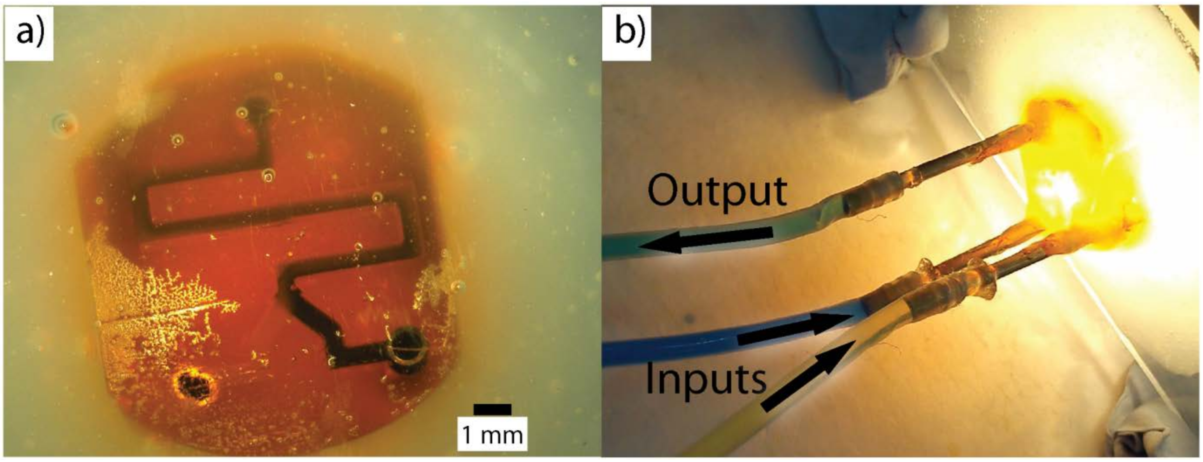

Supplementary Figure S1: Flow mixing millifluidic device, channels have a 400µm cross-section. 2 Aqueous dyes are used.

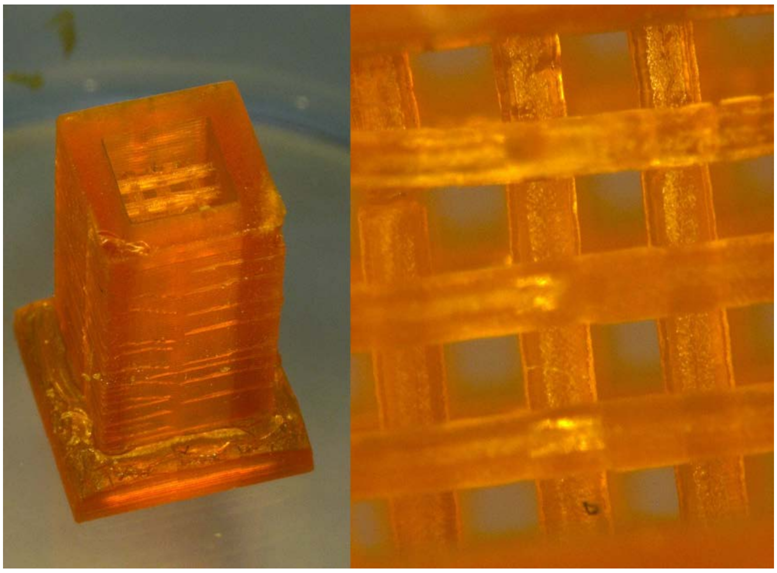

Supplementart Figure S2: A scaffold structure featuring 200µm struts in a 1cm tall unit.

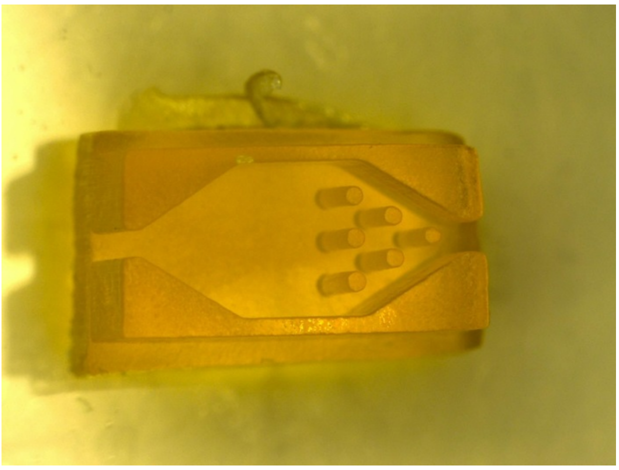

Supplementary Figure S3: “Bagatelle” structure

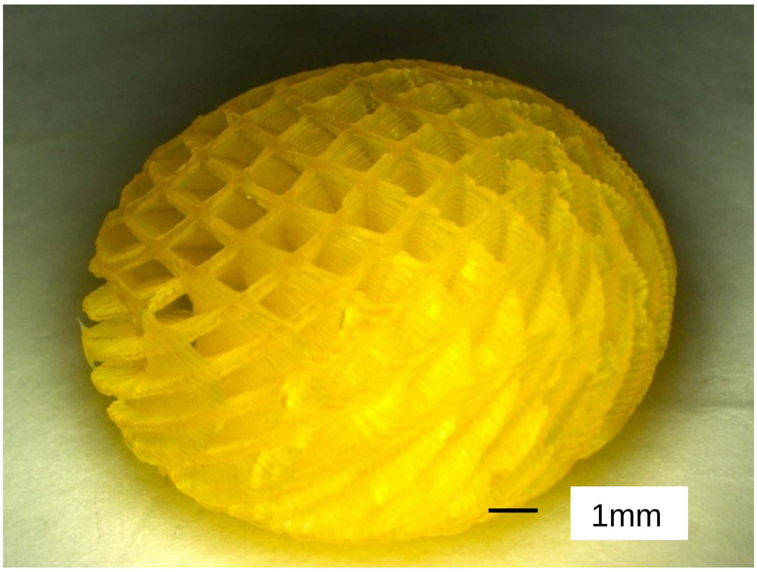

Supplementary Figure S4: Sphere with twisting internal structure

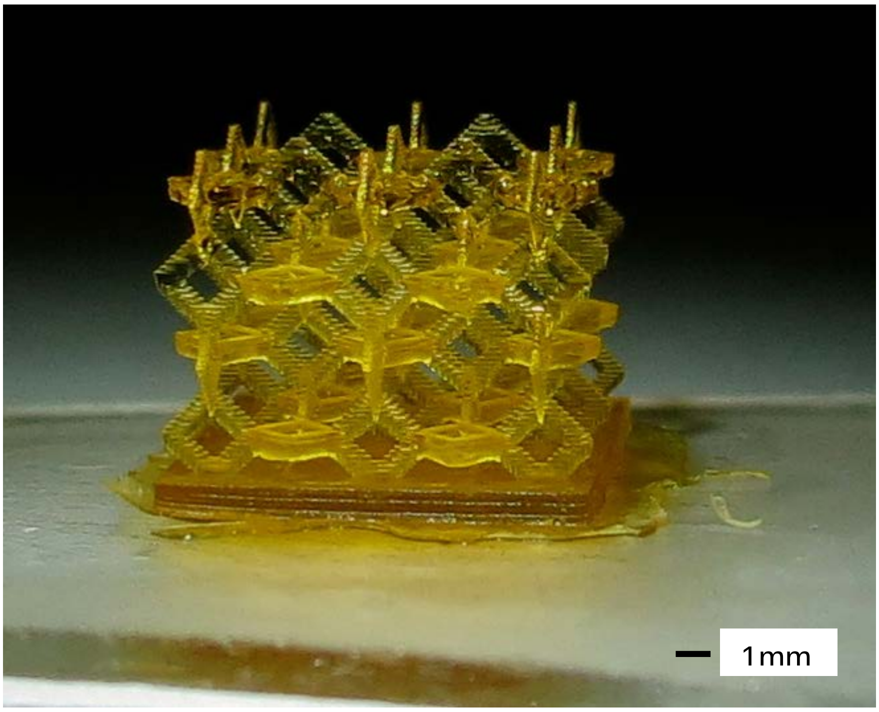

Supplementary Figure S5: A photograph of an intricate lattice structure.

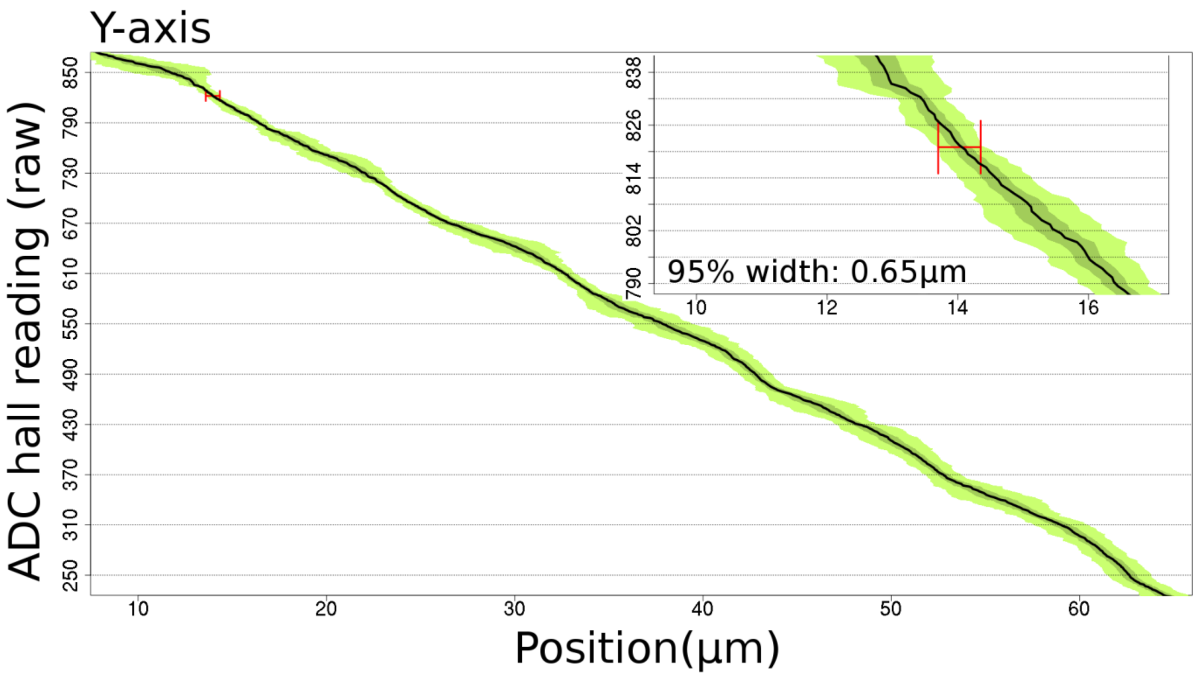

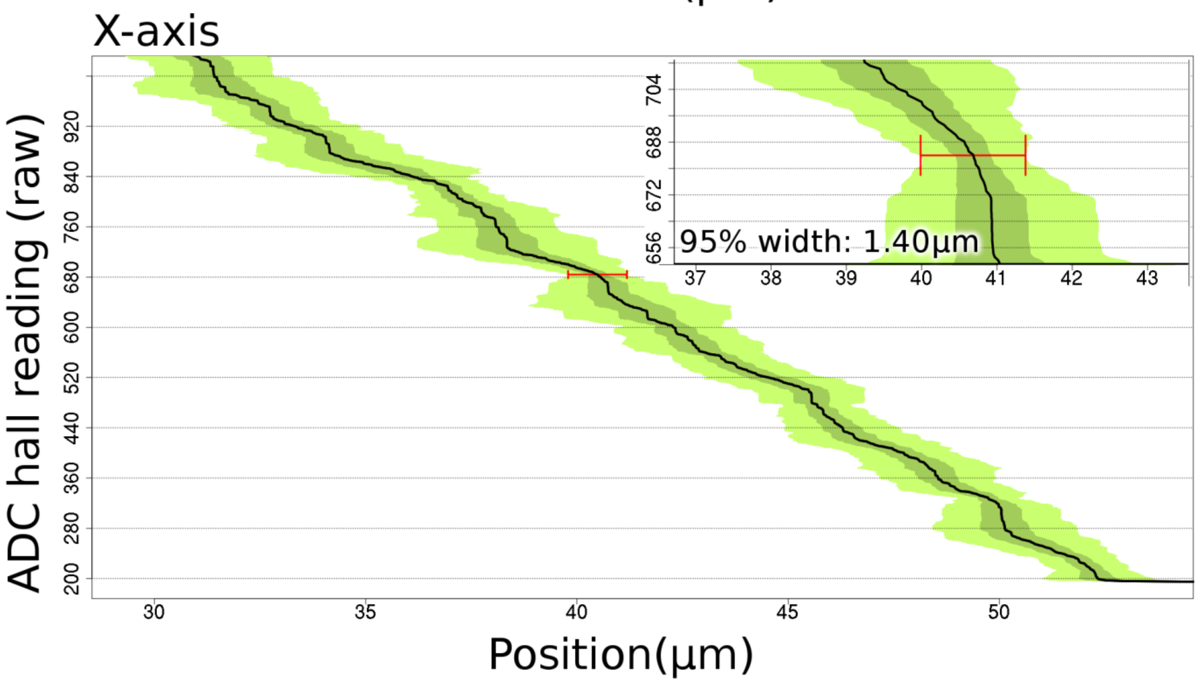

Supplementary Figure S6: Variation in the Hall sensor reading, at and around the home position for Y-axis (top) and X-axis (bottom). The variation in the reading give a positioning error of 1µm.

Calibration

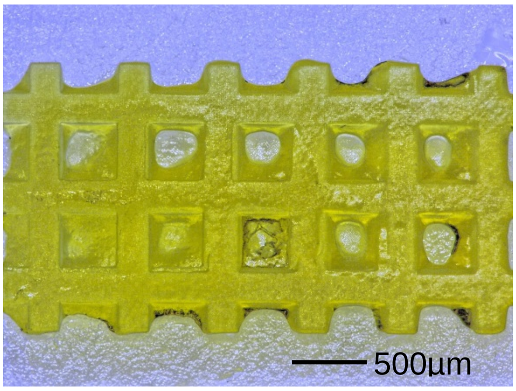

Supplementary Figure S7: Calibration of DMD pixel size to millimetres in the print. X and Y distances where measured with a Keyence digital microscope images analysis.

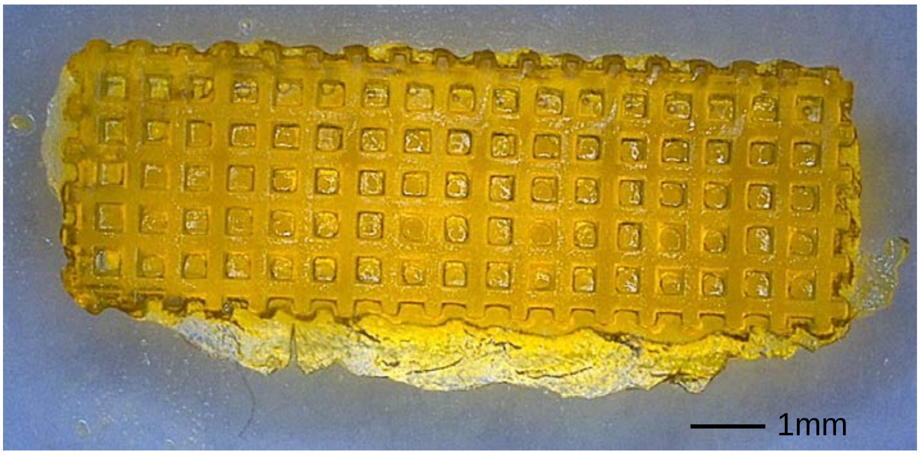

Supplementary Figure S8: Calibration of X stage microsteps to millimetres. Calibration was adjusted so that the projection module moved one image's width.

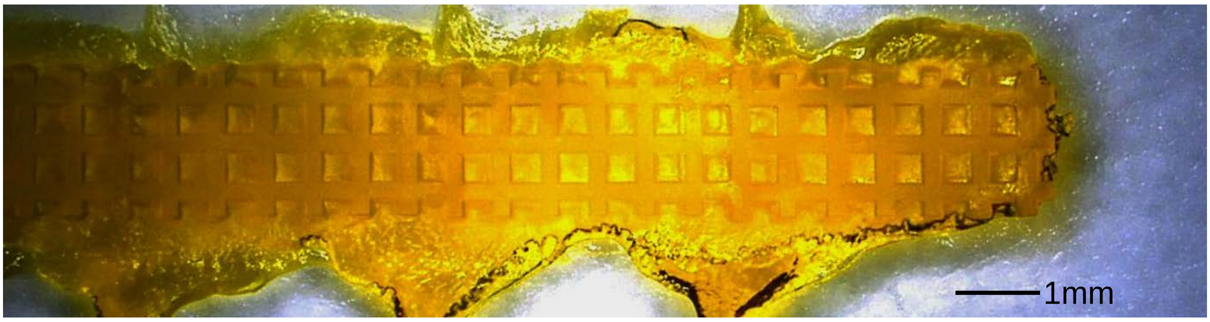

Supplementary Figure S9: Calibration of Y stage microsteps to millimetres. Calibration was adjusted so that the projection module moved one image's height.

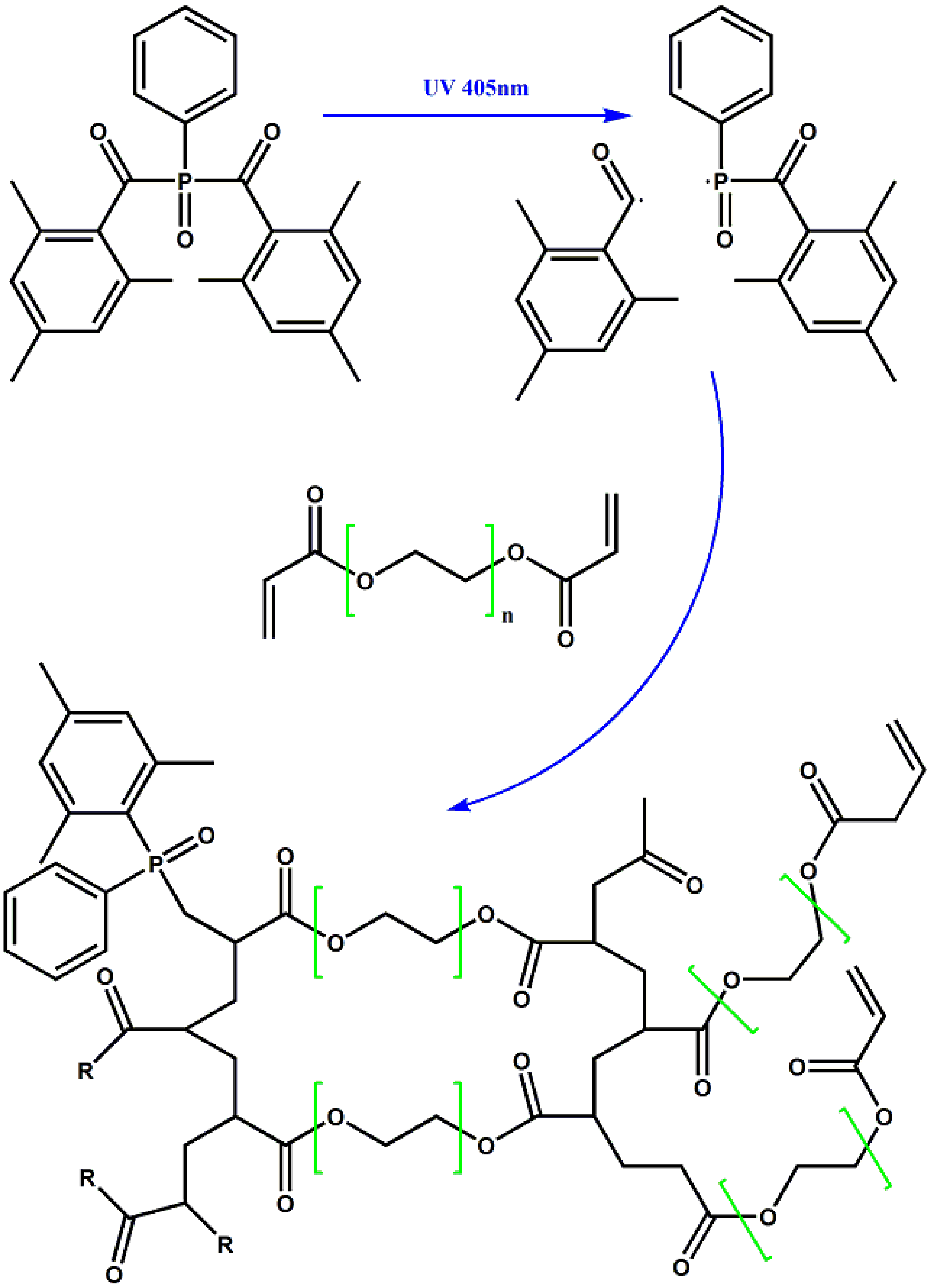

Supplementary Figure S10: Scheme of polymerisation reaction.

Build Software

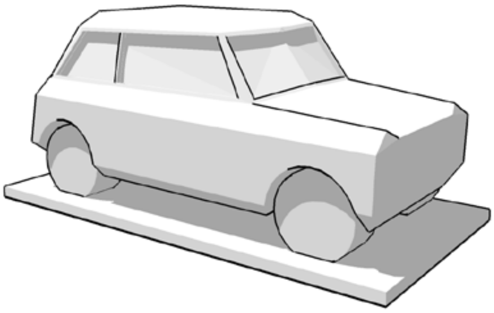

Supplementary Figure S11: Example STL file to be printed.

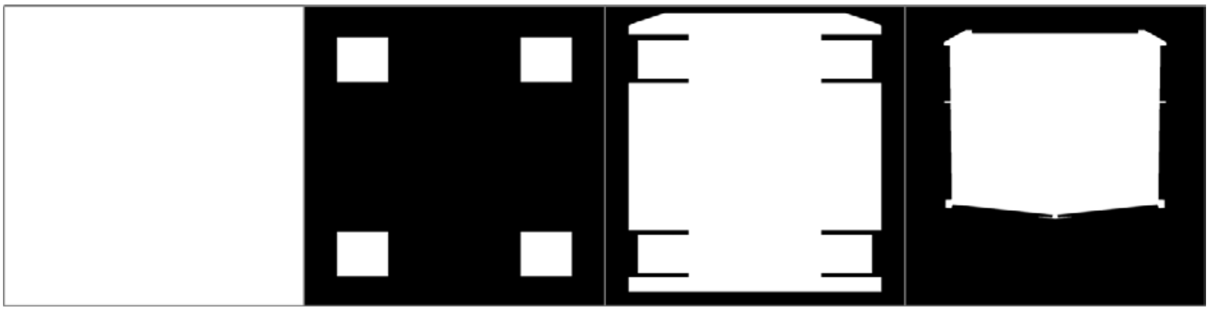

Supplementary Figure S12: Example PNG images created by horizontal slicing of the stl model. Owing to the mapping of pixels on the projector, our image files are stretched by a factor of two in x.

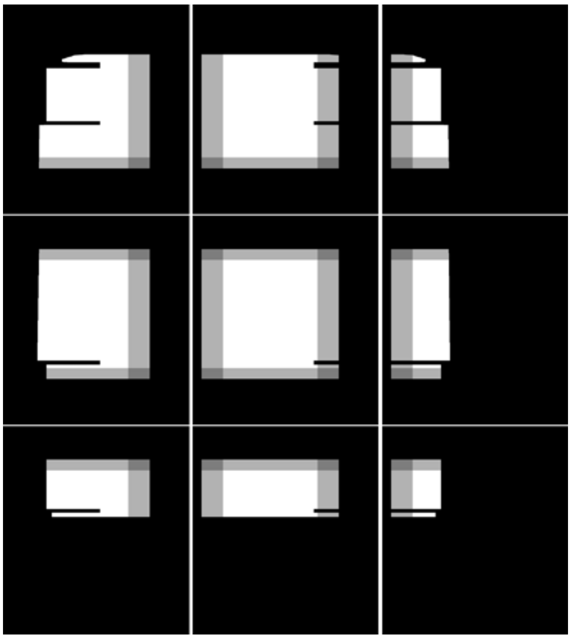

Supplementary Figure S13: The image is subdivided into individual exposures.

Control Software

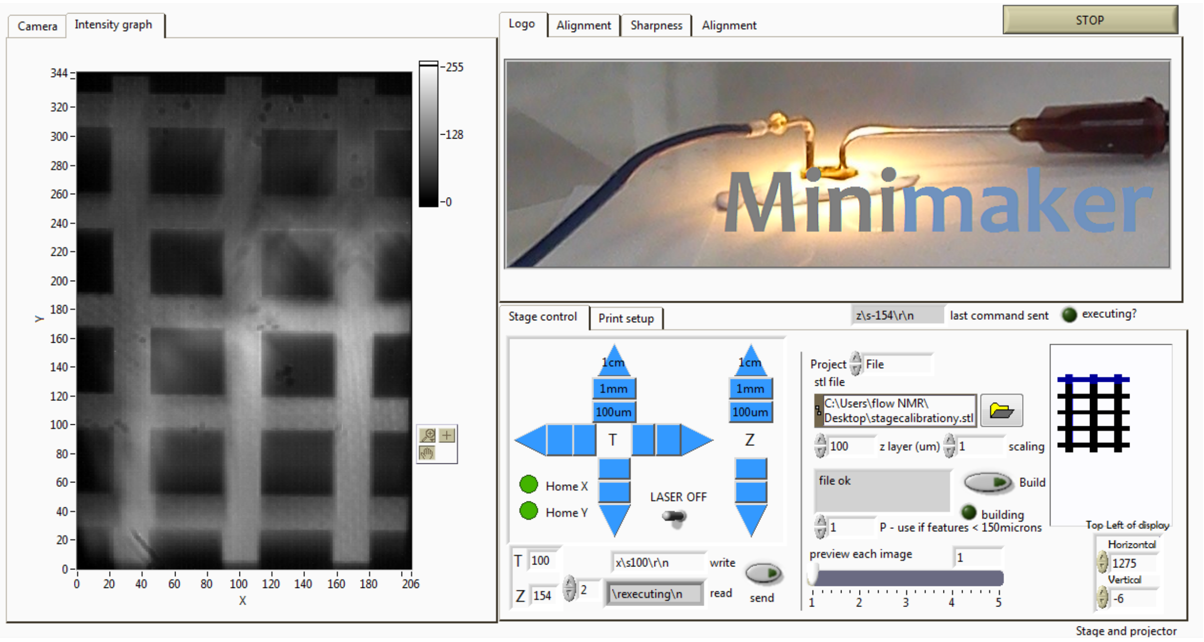

Supplementary Figure S14: Control software user interface (Stage control tab): Three axis stage controls, laser control, and build settings on right. Live camera images displayed on left.

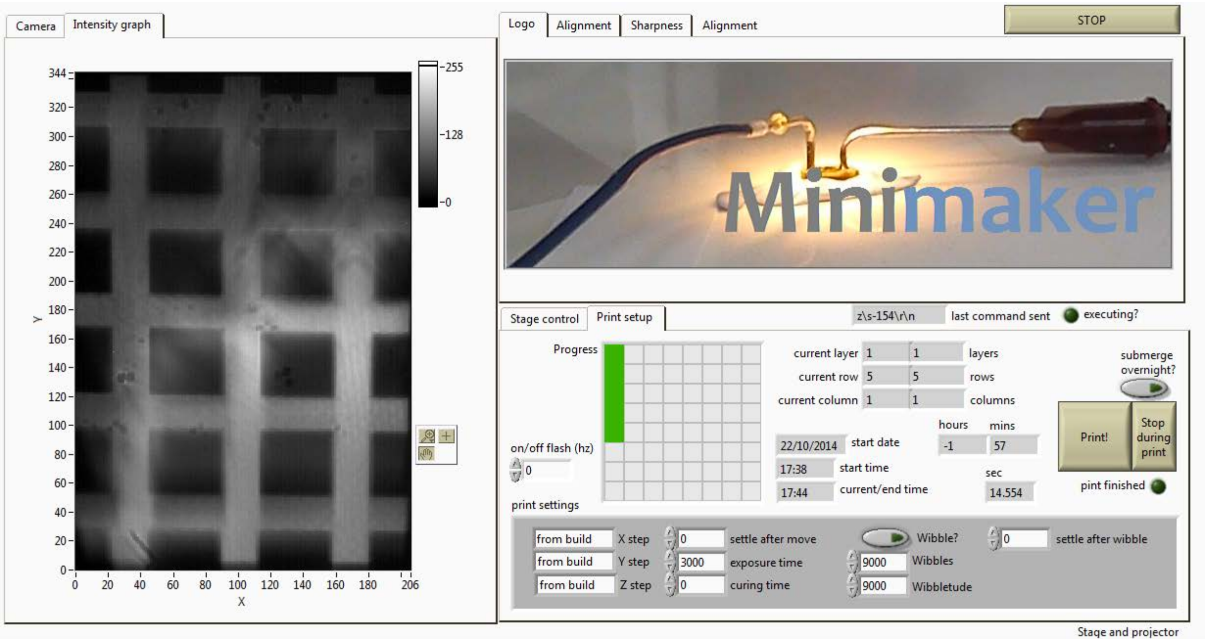

Supplementary Figure S15: Control software user interface (Print set up tab): Print settings such as exposure time controls, start print, and progress indicators.
